# Geospatial estimation of reproductive, maternal, newborn and child health indicators: a systematic review of methodological aspects of studies based on household surveys

**DOI:** 10.1186/s12942-020-00239-9

**Published:** 2020-10-13

**Authors:** Leonardo Z. Ferreira, Cauane Blumenberg, C. Edson Utazi, Kristine Nilsen, Fernando P. Hartwig, Andrew J. Tatem, Aluisio J. D. Barros

**Affiliations:** 1 International Center for Equity in Health, Universidade Federal de Pelotas, Pelotas, Brazil; 2 Post-Graduate Program in Epidemiology, Universidade Federal de Pelotas, Pelotas, Brazil; 3 WorldPop, Geography and Environmental Science, University of Southampton, Southampton, UK

**Keywords:** Geospatial modeling, Small area estimation, Reproductive health, Maternal health, Newborn health, Child health, Low- and middle-income countries, Household surveys

## Abstract

**Background:**

Geospatial approaches are increasingly used to produce fine spatial scale estimates of reproductive, maternal, newborn and child health (RMNCH) indicators in low- and middle-income countries (LMICs). This study aims to describe important methodological aspects and specificities of geospatial approaches applied to RMNCH coverage and impact outcomes and enable non-specialist readers to critically evaluate and interpret these studies.

**Methods:**

Two independent searches were carried out using Medline, Web of Science, Scopus, SCIELO and LILACS electronic databases. Studies based on survey data using geospatial approaches on RMNCH in LMICs were considered eligible. Studies whose outcomes were not measures of occurrence were excluded.

**Results:**

We identified 82 studies focused on over 30 different RMNCH outcomes. Bayesian hierarchical models were the predominant modeling approach found in 62 studies. 5 × 5 km estimates were the most common resolution and the main source of information was Demographic and Health Surveys. Model validation was under reported, with the out-of-sample method being reported in only 56% of the studies and 13% of the studies did not present a single validation metric. Uncertainty assessment and reporting lacked standardization, and more than a quarter of the studies failed to report any uncertainty measure.

**Conclusions:**

The field of geospatial estimation focused on RMNCH outcomes is clearly expanding. However, despite the adoption of a standardized conceptual modeling framework for generating finer spatial scale estimates, methodological aspects such as model validation and uncertainty demand further attention as they are both essential in assisting the reader to evaluate the estimates that are being presented.

## Background

Reproductive, maternal, newborn and child health (RMNCH) is central to the Sustainable Development Goals (SDG) agenda for 2030 given its potential for improving health and quality of life of current and future generations as summarized by the motto “survive, thrive, transform” adopted by the Every Woman Every Child initiative [1]. Despite progress in the area, with the increase in coverage of several indicators, there is yet much to be achieved [2]. Planning and implementation of essential health interventions, delivered by supporting organizations and governments, is mainly done at small administrative divisions such as districts, states, provinces, regions or counties [3]. This requires geographically disaggregated information, which enables more precise adjustment of policies and targeting of resources [4].

Information on RMNCH indicators is predominantly obtained from national health surveys in low- and middle-income countries (LMIC), which offer standardized and reliable estimates [5]. Still, most surveys are usually designed to provide representative estimates at the largest administrative divisions as further disaggregation would require larger sample sizes [6]. Different estimation methods are required since direct estimation of lower administrative units in these surveys is highly imprecise. Geospatial approaches have been widely used for estimating RMNCH outcomes for small areas using georeferenced survey data. These methods derive indirect estimates from statistical models by ‘borrowing strength’ across space or from supplementary data, such as geospatial variables, censuses and administrative records [7]. However, censuses are carried out every 10 years or more in LMICs and administrative records are often incomplete, of poor quality or unavailable. Therefore, geospatial variables (information that is continuous across space, often retrieved from satellites or spatial interpolation), have been frequently used as supplementary data given their availability, timeliness, and reliance. The literature often uses the terms model-based geostatistics, small area estimation and (geo)spatial modeling interchangeably as model-based approaches to derive estimates for small geographies assisted by supplementary data.

Despite the rapid increase in the use of geographic information systems in RMNCH over the past decades, only a few studies have attempted to summarize these efforts. Two of them presented a broad review of spatial analyses in RMNCH [8] and health surveys in Sub-Saharan Africa [9], while one study focused on malaria transmission modeling [10]. Lastly, Rahman [11] carried out a review focusing on the methods used for estimation. To our knowledge, no study has comprehensively evaluated the most important methodological aspects for geospatial estimation of RMNCH indicators in LMICs. This assessment is necessary to identify approaches currently being used, their strengths and limitations and to help inform and improve future studies. Also, since these methodologies are relatively complex, non-specialists may struggle to evaluate and correctly interpret such studies. Therefore, this study aims to discuss the core methodological aspects of geospatial estimation, including any specificities employed for each RMNCH outcome, in studies focused on producing fine spatial scale estimates. In addition, we aim to enable non-specialist readers to critically evaluate and interpret these studies.

## Methods

### Conceptual framework

**Fig. 1:**
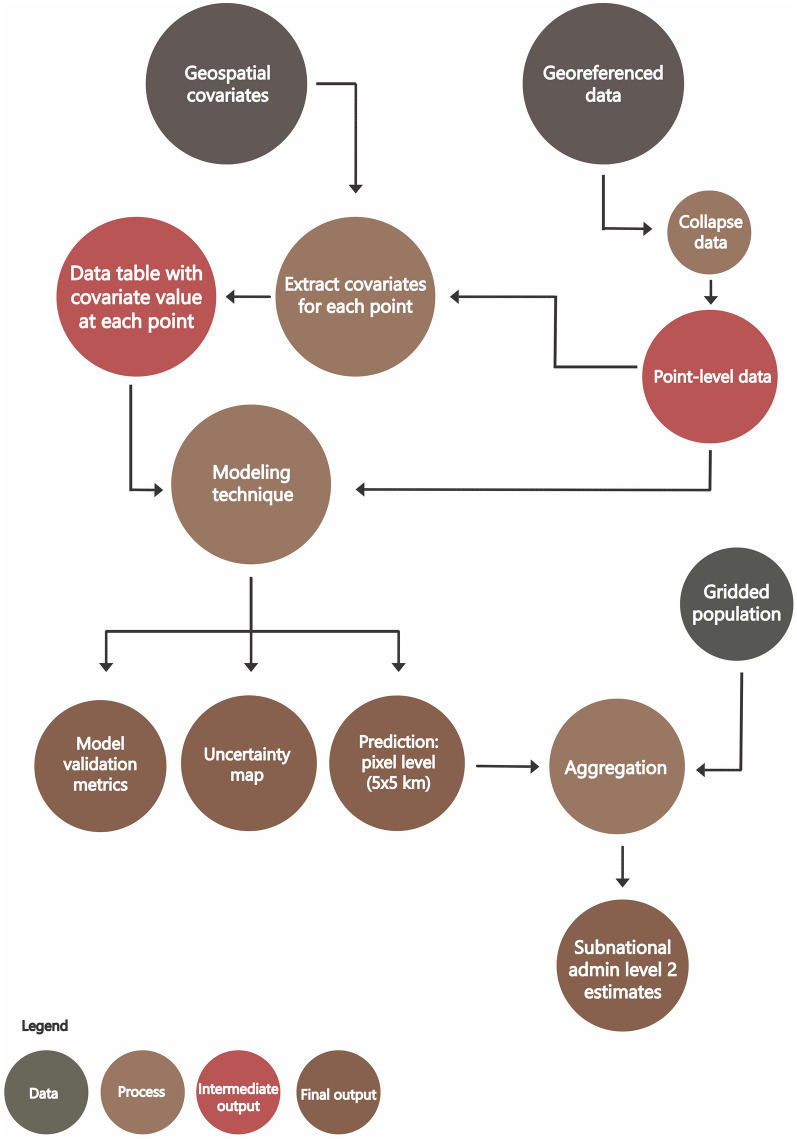
Overview of a geospatial estimation process (adapted from Mayala et al. [12])

The structure and methodological aspects discussed in the review are guided by a standard modeling framework, adapted from Mayala et al. [12] and presented in Fig. 1. This conceptual framework is widely adopted in the literature and geospatial estimation studies, as it defines the flow of the modeling process. The use of the conceptual framework is not part of the eligibility criteria and has no effect on the selection of the studies.

### Search strategy

Two independent reviewers carried out the same search strategy on August 28th 2020, screened and extracted the characteristics of the studies. Medline, Web of Science, Scopus, SCIELO and LILACS electronic databases were searched for studies based on survey data which applied geospatial approaches to estimate RMNCH outcomes in LMICs.

The search strategy consisted of a combination of health and geospatial keywords. The keywords “*health”* and “*epidemiology”* were used to define a broad health construct, rather than focusing on RMNCH outcomes, to increase the sensitivity of the search. For geospatial approaches, keywords were: “*geostatistical”*, “*geo*-*statistical”*, “*spatial modeling*”, “*spatial modelling*”, “*high*-*resolution mapping*”, “*geospatial”*, “*small area estimation*”, “*small area estimates*” and “*spatial interpolation*”. The complete keywords combination using logical operators is provided in Additional file 1. No restrictions on language or publication date were applied. In addition to the electronic databases, reference lists of the selected articles were searched for additional eligible studies not detected by the initial search strategy.

Articles retrieved from the search strategy were combined using Mendeley and exported to Rayyan, a web application for systematic reviews, for screening [13]. Initial duplicates were automatically removed in Mendeley, and the remainder were manually removed using Rayyan. The protocol for the systematic review was registered on PROSPERO (ID: 206323). This review follows the guidelines from PRISMA, and the checklist is provided in Additional file 2.

### Eligibility criteria

To be eligible, studies must have fulfilled all the following criteria:

1. Carried out model-based geospatial approaches to obtain more geographically precise estimates than allowed by direct estimation due to insufficient sample size or to lack of representativeness;
2. Focused on RMNCH outcomes: coverage or impact indicators relevant to public health policies for women in reproductive age (15 to 49 years) or children aged < 5 years. Studies covering a broader age range for children but including the desired ages were also eligible;
3. Outcomes had to be measured in LMICs as defined by the 2020 World Bank country-income classification [14];
4. The main source of information were survey data and the minimum geographical coverage was an entire country.

The eligibility criteria were applied to all retrieved studies. Records were independently screened by both reviewers, first assessing titles and abstracts, then by reading the full text of the selected studies.

### Exclusion criteria

Studies that did not estimate measures of occurrence of the coverage or impact outcomes were excluded.

### Data extraction and quality assessment

We developed a Microsoft Excel spreadsheet to extract relevant characteristics of the selected studies based on ten pre-selected studies and on expert opinion. Then, each reviewer manually extracted the information from all the selected studies separately and the spreadsheets were compared later with disagreements dealt by consensus. The extracted characteristics, details, and guidance on how the spreadsheet was filled can be found in Additional file 3. Quality of the studies was assessed using Joanna Briggs Institute checklist for prevalence studies [15] and presented in Additional file 3.

## Results of the literature search

**Fig. 2:**
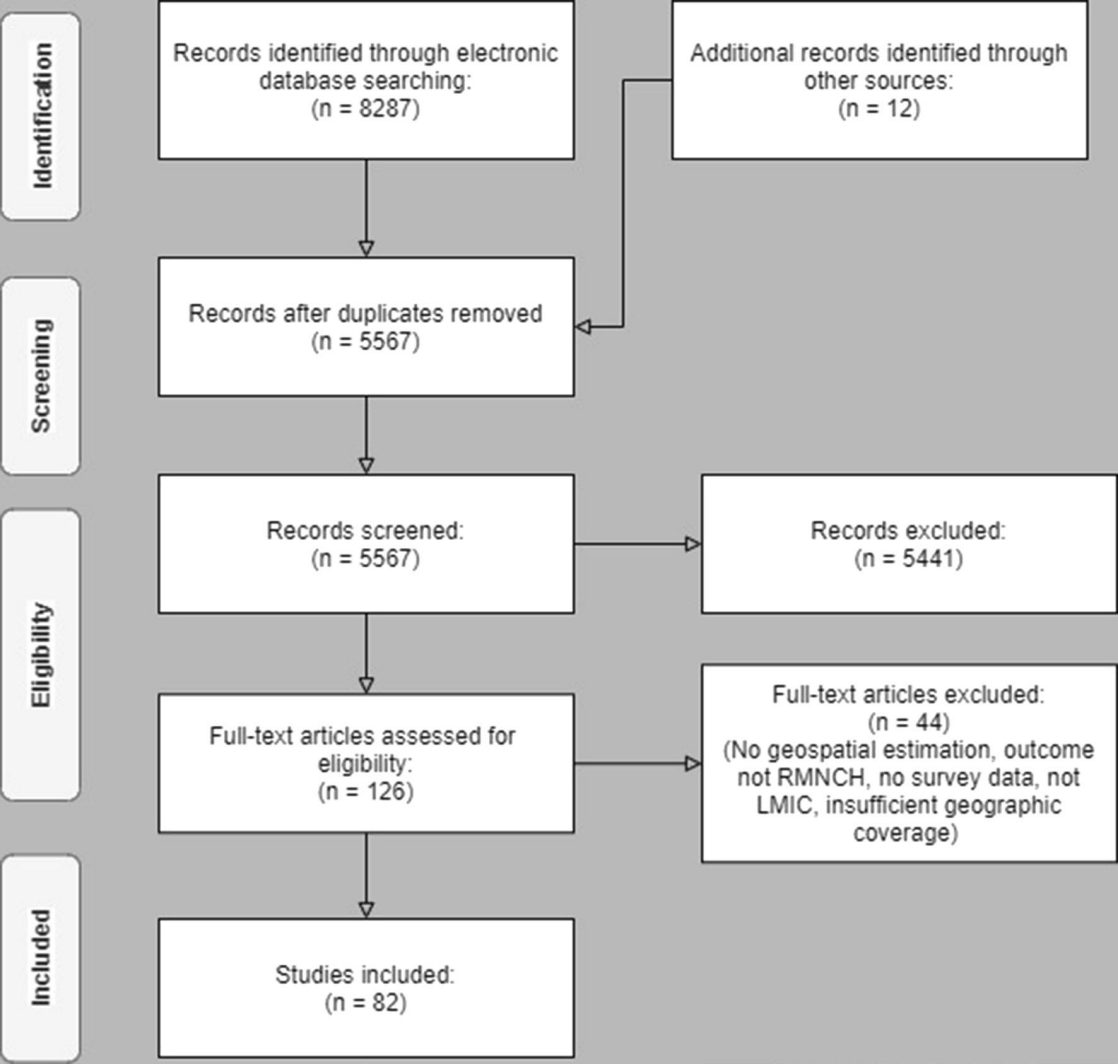
Flow diagram of study selection

After removing duplicates, 5567 records were identified for title and abstract screening, resulting in 126 selected articles. After full-text assessment, another 44 studies were removed yielding a total of 82 studies included in this review (Fig. 2). Several studies using the methods of interest, but estimating outcomes not considered to be RMNCH or covering age ranges outside our focus were not included in the review. The earliest studies identified were carried out in 2000, but the field grew steadily since 2016, comprising over 50% of the included studies (Additional file 1). The following sections discuss methodological aspects and outcomes. Due to the large number of studies reviewed, the following sections do not cite all studies in their respective categories. Details from each study are provided in Additional file 3.

## Methodological aspects

Ideally, models built to predict unobserved data aim to minimize prediction errors, bias and overfitting of the data. Certain decisions are taken in each step of the process and presenting them in an organized and clear fashion is essential to allow readers to assess how reliable the estimates are. Based on the conceptual modeling framework presented above, we discuss the most important steps of geospatial estimation and details on how studies are reporting crucial information for their interpretation. These steps include data sources, covariates, modeling techniques, resolution, model validation and uncertainty.

### Data sources

RMNCH outcomes in LMICs are often estimated using data from national health surveys. The Demographic and Health Surveys (DHS), a series of nationally representative household surveys conducted in over 85 countries [18], was the leading source of RMNCH information used in 59 of the 82 selected studies (72%). Further data sources include the Multiple Indicator Cluster Surveys [16], Performance Monitoring for Action [17], country-specific health surveys, censuses, and community surveys (main source of information for malaria).

DHS data are available at both administrative (or areal) level (e.g. regions, districts, provinces) and point level, i.e. the centroids of each primary sampling units (or survey clusters). The main difference between areal and point data is the aggregation of the data. While areal data are always summaries of individual level data, points can have both individual and aggregated information. For privacy reasons, DHS adds noise to their GPS coordinates, displacing them in a radius of up to 2 km for urban areas, up to 5 km for rural areas, and up to 10 km in 1% of the rural points. To account for this variation, DHS recommends drawing a buffer around each coordinate and averaging the neighboring values instead of using a precise match [19]. Despite that, only 16 of the 36 studies that used point-level DHS data reported taking steps regarding the displaced coordinates. Gething et al. [20] described the impact of the displacement as modest, overall, but varying between outcomes and locations.

### Geospatial covariates

Geospatial variables or covariates are sources of information from determinants or proxies of determinants that are used as predictors in geospatial estimation for any given outcome. Obtaining and processing covariates is the most challenging and time-consuming step of the geospatial estimation process since the availability of this information is often limited to raw satellite indices, previous work, and a few initiatives. Covariates are used in the model for estimation and prediction and must be prepared accordingly. For estimation, each covariate information is extracted to the survey cluster location (or the available administrative level for areal models) and provided to the model along with the outcome. After model fitting, for prediction, a surface layer for each covariate is required at the desired resolution. Since these covariates often come from different data sources, aggregation is required when resolution is too high (e.g. satellite information) and interpolation when resolution is too low (e.g. creating surface layers from survey cluster coordinates).

The average number of covariates used across all studies was 9, ranging from 0 to 40. A total of 15 studies did not include any information on covariates into their models. We classified the covariates into seven groups: agriculture and livestock, climate, health-related interventions and outcomes, remoteness, satellite indices, sociodemographic, and topography and land cover. Covariates related to topography and land cover were the most common predictors found in 59 studies, followed by sociodemographic characteristics (53 studies), climate (43 studies) and remoteness (43 studies), as presented in Table 1. Additional file 3 provides the complete list of covariates for each study and their respective classifications.

**Table 1:** Summary of the characteristics for the selected studies

	All studies	Study outcomes^a^				
		Malaria	Child mortality	Malnutrition	Vaccination	Other outcomes
Number of studies	82	34	14	11	8	19
Covariates^a^						
Agriculture and livestock	17	3	3	5	4	5
Climate	43	28	5	5	4	5
Health-related interventions/outcomes	24	9	5	5	1	7
Remoteness	43	19	7	5	5	11
Satellite indices	19	4	5	3	4	7
Sociodemographic	53	17	6	11	6	17
Topography/land cover	59	30	7	10	4	12
No covariates	15	4	7	1	2	2
Geographic coverage						
Single country	50	26	6	7	2	9
Multi-country	32	8	8	5	6	10
Temporal component						
No	46	19	2	8	7	11
Yes	36	15	12	4	1	8
Spatial resolution^a^						
Less than 5x5km	23	18	0	1	3	2
5x5 to 10x10km	20	6	5	5	3	4
Lower admin. level	30	2	8	6	1	14
Not reported	12	10	1	1	0	0
Uncertainty^a^						
Standard deviation map	14	6	1	1	5	2
Interval map/table	28	12	8	2	0	10
Relative map	7	0	2	3	0	2
Other metrics	13	9	2	0	1	1
Not reported	22	7	3	6	2	4
Modeling technique^a^						
Bayesian–MCMC	35	24	3	3	3	2
Bayesian–INLA	28	4	7	6	3	12
Classical GLM	17	5	2	2	2	6
Spatial interpolation	2	0	1	1	0	0
Ensemble models	12	1	5	4	1	5
Out-of-sample pred.						
Cross-validation	22	3	7	5	4	6
Hold-out	24	18	2	1	0	4
Not reported	36	13	5	6	4	9
Model fit metrics^a^						
Bias	34	12	7	6	4	9
RMSE/MSE	30	3	7	6	6	12
Coverage	24	8	6	4	4	5
DIC/AIC	19	6	3	3	1	6
MAE	16	7	2	3	2	3
Correlation	15	11	0	2	1	2
Other metrics	31	15	4	3	1	9
None reported	11	5	2	3	1	1

^a^These characteristics allow studies to be classified in more than one subgroup

*MCMC* Markov Chain Monte Carlo, *INLA* Integrated Nested Laplace Approximation, *GLM* Generalized Linear Models, *RMSE* Root Mean Squared Error, *MSE* Mean Squared Error, *DIC* Deviance Information Criterion, *AIC* Akaike Information Criterion, *MAE* Mean Absolute Error

The optimal number of covariates chosen as predictors, in order to optimize the refined estimation of the outcomes of interest, is a frequent topic of discussion. The principle of parsimony endorses the use of few and strong explanatory covariates to prevent overfitting the data. However, strong predictors are rarely available and insufficient covariate information may lead to model misspecification. This effort in finding the balance reinforces the importance of model validation (discussed later in the paper).

### Modeling techniques

Once outcome and covariate information are prepared, they are passed to the chosen modeling technique, including random coefficients to account for spatial correlation and, sometimes, temporal correlation. The Bayesian approach was predominant in the selected studies, as 62 of the 82 studies were based on Bayesian hierarchical models (Table 1). The main conceptual difference, in comparison to the frequentist approach, lies on how the Bayesian framework interpret probabilities. In a frequentist framework, only repeatable events have probabilities, while Bayesian frameworks can assign probabilities to any event [21]. Since Bayesian frameworks also consider the distribution of its parameters, they generate complex posterior distributions, in which exact solutions are often not possible and numerical approximation techniques are required to fit the models. Markov Chain Monte Carlo (MCMC) methods approximate the true posterior distribution by generating dependent samples from it [22]. MCMC can be considered a turning point for Bayesian inference, having been used for model-fitting in 35 of the 62 studies that relied on Bayesian hierarchical models. More recently, Rue and colleagues [23] developed an alternative method called Integrated Nested Laplace Approximation (INLA), which quickly became popular given that it is much faster and yields very similar results compared to MCMC. Despite the first identified studies using INLA being carried out only in 2014, the method has already replaced MCMC in 28 studies. Frequentist estimation was applied in 17 studies through classical generalized linear models. Only two studies used spatial interpolation methods such as kriging [24] and kernel density estimation [25].

Recent studies have started using an ensemble approach, known as stacked generalization, to improve model performance [26]. Briefly, this strategy consists of fitting several models (usually each model uses a different modeling technique), generating intermediate predictions. These predictions are then used as input to a second model. The use of multiple modeling techniques allows any complex non-linear effects of the covariates to be captured, while the final predictions are estimated using a robust, consolidated modeling technique. All 11 studies following this approach [27–37] used a Bayesian hierarchical model fitted using INLA for the final predictions. Only one study relied on ensemble models and did not perform stacked generalization [38].

In addition to borrowing strength from covariance structures through space, spatio-temporal models can also benefit from these structures through time. The inclusion of a temporal component was identified in 36 studies as observed in Table 1. In 34 out of the 36 studies, this approach attempted to evaluate changes over time. These effects were primarily modelled using conditional autoregressive models and stochastic partial differentiation equation as described by Blangiardo et al. [39].

### Resolution

Estimates are typically generated at two different levels of aggregation: grid cells or country’s administrative divisions. At grid cell-level, the entire country is divided into an equally sized grid and predictions are made for each cell individually. A total of 55 out of the 82 studies opted for gridded-estimates (Table 1). Apart from three studies [40–42], the approximate cell size (grid) for all reported resolutions ranged from 1 × 1 km to 10 × 10 km, with 5 x 5 km being the most common one. Smoothed maps were presented by 12 studies without specifying the originally estimated resolution. Estimates for districts, counties, provinces and other low administrative divisions were produced by 30 studies (37%). These administrative level estimates are often produced from the grid level estimates through population-weighted aggregation using gridded population data from, e.g., WorldPop [43]. Only five of the 82 studies presented estimates at both grid cell and administrative levels. There is much discussion regarding the ideal level of aggregation, as it depends on multiple factors including the outcome, the objective of the analysis, how decentralized decision-making is within the country and the trade-off between precision and resolution [41, 44].

### Model validation

A good predictive model is a model capable of reproducing the process that generates the outcome. However, depending on the outcome, available covariates and model specification, its performance can vary substantially [45]. Models should be validated against data that was not used in its construction. Otherwise, the model can learn the data instead of their underlying structure, a phenomenon known as overfitting the data. The simplest choice for out-of-sample predictions is known as the hold-out method, which splits the data into two subsets—a training subset used for model fitting and a test subset used for validation. This approach was found in 29% of the studies (Table 1). However, splitting the data and ensuring geographical representativity in both samples is an overlooked challenge. Seven studies [47–53], though, attempted to overcome this limitation using a declustering method, which gives less weight to observations geographically clustered when drawing the samples [54]. An alternative method for out-of-sample prediction is the n-fold cross-validation, found in 22 studies. The algorithm divides the data in “n” parts of equal size (which can be done in a spatially structured or random manner), leaving one for validation and using the remaining to build the model. This process is repeated until each fold is used for validation and the average of the combined measures is taken. Cross-validation is particularly useful when data is limited and holding out data could compromise the model performance, while the drawback is that it must fit one model for each fold, drastically increasing the processing time. Nearly half of the studies (44%) did not report on any validation method.

Within the out-of-sample data, there are several metrics that can be calculated and reported to assess the validity of the model predictions. As shown in Table 1, bias was the most reported validation measure, present in 34 studies (41%). Bias is the average of the difference between the observed and the predicted value. The magnitude of the prediction errors was often reported using the root mean squared error (RMSE) or the mean absolute error (MAE), found in 30 and 15 studies, respectively. RMSE and MAE are both positive values indicating how much, on average, the model predictions differ from the observed results. They differ on how deviations are handled: RMSE takes deviations squared while MAE ignores the signal. This makes RMSE more susceptible to the impact of high magnitude prediction errors (such data points are often referred to as outliers) [46]. A total of 19 studies presented Deviance Information Criterion (DIC) or similar metrics during model selection or validation. While DIC is useful for model selection, it has no direct interpretation and cannot be used to compare different studies. Additionally, several studies reported the achieved coverage within credible intervals [24] and the correlation between predicted and observed values [16].

### Presentation of uncertainty

As much as the most precise estimates are desirable, there is always a degree of uncertainty in predictions made. While geographically disaggregated point estimates are easily interpretable when presented in a map, the related uncertainty is much harder to present in an intuitive way. Uncertainty is a complex multi-layer concept and it exists in every step from data collection to the modeled estimates. For the sake of this study, we considered uncertainty as the measures of variability associated to the estimates, since a complete definition includes measurable and unmeasurable components, sampling, modeling strategies, and is out of the scope of this study.

Visualization approaches to present uncertainty in a clear, comprehensive, and interpretable manner are still to be proposed. Bayesian models, for instance, produce full posterior estimates that can be summarized in multiple ways. However, there is no visualization approach that can fully address the challenges of communicating and using uncertainty and, as a result, the literature clearly lacks standardization.

Options for presenting uncertainty are tied to the chosen resolution. At the administrative level, where there is a smaller number of divisions, uncertainty can be described using maps or tables. On the other hand, grids of high resolution can only be represented in maps due to the large quantity of estimates. Uncertainty intervals and standard deviation maps and tables were the most common approaches, found in 28 and 15 studies, respectively. There were also seven studies presenting qualitative measures of uncertainty (e.g., low or high uncertainty). Further approaches include: coefficient of variation [55, 56], exceedance thresholds [57, 58], probability of being correctly classified [48, 50, 52] and Coffey-Feingold Bromberg metric [31]. A total of 22 studies (27%) did not present any measure of uncertainty (Table 1). Aside from the numerous ways of expressing uncertainty, it has been exclusively reported in supplementary files of 23 out of the 60 studies (38%) that presented uncertainty, putting its relevance in check.

Maps with the limits of the uncertainty intervals are often presented in two separate figures, demanding more space, and only covering a best–worst case scenario. Some studies use the width of the interval as an alternative, which is limited when the probabilities are close to zero or 1. Standard deviation maps are harder to interpret, especially for non-specialist readers. Lastly, qualitative measures of uncertainty are likely the easiest to interpret, although defining what is low or high uncertainty is arbitrary.

### Key aspects for interpreting geospatial studies

Maps are long used for presenting geographically disaggregated estimates and are often easily interpretable. However, legend scales may be misleading, especially when intervals of different widths are grouped and presented together, or the amplitude is too narrow or too wide. These caveats are particularly important when several maps are presented in sequence and the reader may assume the legend scales are the same.

Every modeled estimate carry assumptions and uncertainties, and several aspects can be observed to assess their reliability. For instance, data sources must provide sufficient information for models to reproduce the occurrence of the outcome. The data must also come from reliable sources and be temporally close to the objective of the study and the covariates used in the process. In the case of multiple data sources and temporal assessment, constant change over time is often an assumption that needs to be taken into consideration.

Models tend to assume the input data are correct, so any estimates from a good predictive model can only be as accurate as the quality of the data sources. As discussed in previous sections, several metrics can be reported and interpreted to evaluate the validity of the predictive model. Bias, the most reported validation measure, indicates whether prediction errors are systematically leaning towards any direction. Therefore, an unbiased estimator should present bias close to zero. However, the scale of the outcome must always be considered when interpreting these measures. For instance, a bias of 1.5 in settings where the average mortality rates are around 3 is huge (50% of the point estimate), but for mortality rates close to 150, the relative importance of the same bias is much smaller (1% of the point estimate). The same applies for interpreting measures of the magnitude of the prediction errors, such as RMSE and MAE. For coverage indicators (bound between 0% and 100%), RMSE or MAE values of 2 indicate the model deviates from the true value, on average, by 2 percentage points.

Incorporating uncertainty in decision-making is often a major challenge. The interpretation of the estimates requires changes in the thought process to consider probabilities rather than an absolute, fixed value. Non-experts tend to depend on heuristics rather than formal statistics when taking decisions [59]. This raises a question on whether considering uncertainty leads to better decisions or simply discredits information in which uncertainty estimates are high [60]. Associated credible intervals can be interpreted as that we are confident (usually 95% confident) that the true estimate is within the interval. Therefore, smaller intervals reduce the probability of our estimate deviating from the true value. The standard error can be roughly interpreted as how precise the sample mean estimate is in relation to the population mean.

## Outcomes

Around 30 different outcomes were estimated using geospatial approaches among the selected studies. We classified them into five groups based on their frequency and similarity: malaria, child mortality, malnutrition, vaccination, and other health-related outcomes. Within each family of outcomes, their specificities are highlighted and the summary of characteristics for all studies and by outcome is presented in Table 1.

### Malaria

Malaria-related studies could be considered the pioneers in RMNCH geospatial modeling with a large contribution to this field. It took nearly a decade for studies of other RMNCH outcomes to start using geospatial estimation to increase the granularity of their available data. The first identified studies are dated to the early 2000’s [61, 62], despite other spatial statistics in the field of malaria having been used for several years before [63]. Malaria is strongly affected by environmental factors. The mosquitoes of the anopheles species require certain climatic conditions to develop themselves and act as transmission vectors for the disease [64]. This geographical dependence along with the burden of the disease led malaria to be the most studied outcome with 34 out of the 82 studies [38, 42, 47–49, 51–53, 56–58, 61, 62, 65–83].

Most of the information for malaria in LMICs comes from combining multiple malariometric surveys conducted at specific locations. Several projects, such as MARA [84] and the Malaria Atlas Project [85], have worked on putting together geo-referenced malaria survey data, allowing researchers to use the pre-processed databases. These surveys were used in 21 of the 34 studies that focused on malaria. Despite concerns over malariometric surveys being carried out only in endemic areas of high prevalence, evidence shows they are well geographically distributed in various settings [47]. A secondary source of information for malaria are nationally representative surveys, either designed for several RMNCH indicators, such as the standard DHS surveys, or focused on malaria as in the Malaria Indicator Surveys, also carried out by the DHS program. A total of 14 studies relied on these surveys.

Although the malaria burden is not limited to children, they are the most affected subset of the population due to the lack of post-infection immunity [86]. Malaria indicators were reported as malaria prevalence, parasitemia risk or number of infected children. Both children under-five and the standardized age range of 2 to 10 years were the most common age subgroups, as observed in 14 and 12 studies, respectively. A few studies presented estimates for other subgroups such as: 6–59 months [79], under-10 years [61], under-16 years [71], 1–10 years [66, 87] and 1–14 years [65].

Different from other outcomes, malaria studies prioritized high-resolution estimates over small administrative units. The only two studies that presented county [58] and regional [72] level estimates also presented estimates at finer resolutions. Single country studies were predominant with 77% of the geographical coverage, while 82% opted for the Bayesian approach for modeling.

### Childhood mortality

Child survival is a central goal of maternal and child health interventions and it is considered both a health indicator and a measure of human development [88]. Reducing child mortality rates is a long-term priority defined by international organizations and highlighted in both the Millennium Development Goals and the SDGs [89]. Even in high mortality settings, the death of a child is a rare event, thus requiring larger samples sizes in comparison to other RMNCH indicators. Among the reviewed studies, 14 were focused on child mortality.

Within child mortality studies, we identified ten studies focusing on all-cause mortality and four studies presenting cause-specific deaths. All ten studies reporting on all-cause childhood mortality estimated under-five mortality rate, while a few studies also presented estimates for neonatal [30, 90] and infant [90, 91] mortality rates. For cause-specific mortality, deaths by malaria [38], diarrhea [32, 35] and lower respiratory infection [33] were studied.

Most studies, 12 out of 14, assessed changes over time—a major focus for mortality—most likely relating to monitoring development goals. In terms of resolution, six studies aimed at reaching smaller administrative units such as districts or counties [35, 55, 92–96], six presented gridded estimates [25, 30, 32, 33, 38, 91] and one employed both approaches [90]. As with all outcomes, Bayesian models were predominant, used in 10 out of 14 studies. Four studies attempted to develop or enhance methods to estimate under-five mortality.

### Malnutrition

Each year, 3.1 million deaths of under-five children are directly attributable to undernutrition in the form of stunting, wasting and micronutrient deficiencies [97], and overweight in children is an increasing problem. A total of 12 studies focused on malnutrition.

The burden of stunting, wasting, underweight and overweight was estimated for the entire African continent [28] and in all LMICs [27, 37]. There were also several single country studies that account for and focus on local specificities as done in Bangladesh [98], Afghanistan [99], Cambodia [100], India [36], Mexico [101] and Ethiopia [24, 102]. Five studies generated estimates at district or province level [98–100, 102], four studies at 1x1km [45], 5x5km [27, 28] and 10x10km [103], and two studies at both 5x5km and administrative level [36, 37]. Six studies modeled their outcomes using Bayesian models through INLA.

Among all outcomes, uncertainty was least reported on studies focusing on malnutrition, available in only half of the studies.

### Immunization

Vaccines save the lives of millions of children every year, and despite being one of the most cost-effective health interventions, many settings have seen coverage levels stall or even decline in recent years [104]. For measles, which is highlighted in six of the seven immunization studies, many outbreaks occurred globally in 2018 and 2019, mainly due to lack of access and anti-vaccination movements [105–107].

Geospatial modeling of immunization started relatively recently, since all identified studies were published from 2015 onwards. Possibly due to being very recent, most of them carried out very comprehensive modeling approaches. Six of the eight studies produced estimates for at least three countries and only two failed to report uncertainty measures. The granularity pursued was also very high, having three studies at 1x1km [108–110], three studies at 5x5km [31, 111, 112] and one at 10x10km [113]. Perhaps due to being the first, Pramanik et al. [114] was the only vaccination study which focused in a single country, aimed at lower administrative units rather than gridded estimates, and one of the two studies that did not report uncertainty measures.

### Other RMNCH outcomes

The use of geospatial approaches to produce estimates for small areas has reached a variety of outcomes. Within reproductive health, we identified four studies focusing on contraception [45, 115–117] and two studies on undesired adolescence pregnancies [118, 119]. From pregnancy to child birth, four studies focused on antenatal care, skilled birth attendance, c-section and post-natal care [40, 41, 120, 121]. Diarrhea [32, 122, 123] and respiratory infections [33] were the focus of a total of seven studies, as they are still among the leading causes of death for children in the poorest countries. One study also attempted to map exclusive breastfeeding [34].

## Conclusions

The field of geospatial estimation focused on RMNCH outcomes is expanding and the number of published studies has increased more rapidly since 2014. Bayesian hierarchical models have taken place as the preferred modeling technique, but this is a continuously evolving area. More recently, ensemble approaches using several different models that are put together with a Bayesian model have been increasingly used and have the potential to become the approach of choice. The main data sources are likely to remain the same, DHS with a special place among national health surveys, especially that they have been putting a lot of effort in providing geolocated covariates available already harmonized with the surveys [125].

Geospatial models are complex and tend to produce a large number of estimates. Therefore, a validity assessment of how assumptions hold, the estimates precision and the model mean error should always be done and presented. These characteristics should be evaluated out-of-sample using one of the several approaches proposed in the literature, with cross-validation being the most efficient in terms of data use. However, with such complex models, fitting a model repeatedly can demand considerable processing power. Nonetheless, this is a key step to show that the results presented are stable and represent the underlying process in study. Model validation needs to be clearly presented both in terms of how it was done and its results. In our review, a considerable number of studies failed to present clear and convincing model validation—36 out of 82 —what makes the results much harder to interpret.

Other important aspects of geospatial modeling are the resolution of the estimates and how these are presented in terms of both point estimates and their uncertainty. The objective of the work is central to choosing the resolution or the type of aggregation to be used. A study describing the spatial distribution of an outcome or showing associations with geographical aspects can present very high-resolution estimates. On the other hand, if the aim is to support health policy decisions, estimates matching health districts, or geographical units where policies and programs are decided and implemented, are likely to be much more useful. The presentation of estimate uncertainty is also essential. However, we have not identified in the literature a clear and robust approach, as this represents a real challenge. Different measures of uncertainty have been used, as well as a variety of approaches of presentation – from simple to complicated. Given its importance, it seems to us that simpler and more direct visualization approaches could be used in the main body of the paper, while full results could be reported in the supplementary material.

As a final comment, given the often-large number of maps and diagrams presented, special attention has to be devoted to comparability of the scales used, color schemes, and even the map projections. The results need to be presented in an intuitive and understandable fashion so that non-specialists can grasp and make use of such relevant estimates. Authors need to put as much effort in the clarity of their presentations as they invest in the complex process of geospatial estimation.

This study covers the main methodological aspects that are part of a standard conceptual modeling framework adopted by the literature. However, many details that are lightly discussed here could be the focus of further studies such as a thorough evaluation and comparison of modeling techniques, covariates, and uncertainty. In addition, concern should be raised on how far these models can be extended, given the expansion of the field to over 30 different outcomes. Since predictions are based on space and time correlation and explanatory variables, producing fine spatial scale estimates may not be feasible for all outcomes [45].

The authors encourage future studies focused on modeling RMNCH outcomes using geospatial approaches to make uncertainty presentation and model validation as an integral part of their studies. In light of the issues of handling uncertainty, incorporating it in the discussion of results could assist readers in their interpretations and facilitate the practical application of geospatial approaches for policy making towards improving RMNCH in LMICs.

## Supplementary information

**Additional file 1.** Search strategy and decisions for each quality criteria.

**Additional file 2.** PRISMA checklist.

**Additional file 3.** Methodological aspects of the reviewed studies.

**Additional file 4.** Decisions behind each screened study.

## Data Availability

Not applicable.

## Abbreviations

RMNCH: Reproductive, maternal, newborn and child health
LMIC: Low- and middle-income countries
DHS: Demographic and Health Surveys
SDG: Sustainable Development Goals
MCMC: Markov Chain Monte Carlo
INLA: Integrated Nested Laplace Approximation
GLM: Generalized Linear Models
RMSE: Root Mean Squared Error
MSE: Mean Squared Error
MAE: Mean Absolute Error
DIC: Deviance Information Criterion
AIC: Akaike Information Criterion
