# Supplementary material for: Geospatial estimation of reproductive, maternal, newborn and child health indicators: a systematic review of methodological aspects of studies based on household surveys
Source: Int J Health Geogr. 2020 Oct 13;19:41. doi: 10.1186/s12942-020-00239-9 (PMC7552506; doi:10.1186/s12942-020-00239-9)
Supplement: Supplementary file 1 — Additional file 1. Search strategy and decisions for each quality criteria. [file 12942_2020_239_MOESM1_ESM.docx]

# Search strategy

## Search strategy for MEDLINE, LILACS, Web of Science and Scielo

(health OR epidemiology) AND (geostatistical OR geo-statistical OR “spatial modeling” OR “spatial modelling” OR “high-resolution mapping” OR geospatial OR “small area estimation” OR “small area estimates” OR “spatial interpolation”)

## Search strategy for Scopus

TITLE-ABS-KEY ( ( health OR epidemiology ) AND ( geostatistic OR "geo statistic" OR "spatial modeling" OR "high-resolution mapping" OR geospatial OR "small area estimation" OR "spatial interpolation" ) ) AND ( LIMIT-TO ( DOCTYPE , "ar" ) OR LIMIT-TO ( DOCTYPE , "re" ) ) AND ( EXCLUDE ( SUBJAREA , "AGRI" ) OR EXCLUDE ( SUBJAREA , "EART" ) OR EXCLUDE ( SUBJAREA , "MATH" ) OR EXCLUDE ( SUBJAREA , "ENGI" ) OR EXCLUDE ( SUBJAREA , "BUSI" ) OR EXCLUDE ( SUBJAREA , "VETE" ) OR EXCLUDE ( SUBJAREA , "CHEM" ) OR EXCLUDE ( SUBJAREA , "ARTS" ) OR EXCLUDE ( SUBJAREA , "PHYS" ) OR EXCLUDE ( SUBJAREA , "PSYC" ) OR EXCLUDE ( SUBJAREA , "NEUR" ) OR EXCLUDE ( SUBJAREA , "ENER" ) OR EXCLUDE ( SUBJAREA , "CENG" ) OR EXCLUDE ( SUBJAREA , "MATE" ) OR EXCLUDE ( SUBJAREA , "DENT" ) )

# Decisions on quality assessment criteria

**Were study participants sampled in an appropriate way?** Studies using several data sources in which most of the data come from structured national health surveys were classified as ‘yes’.

**Was the sample size adequate?** All studies were classified as ‘Not applicable’ because the rationale behind small area estimation assumes insufficient sample sizes for direct estimation.

**Were the study subjects and the setting described in detail?** Studies covering multiple countries were classified as ‘Not applicable’.

**Was data analysis conducted with sufficient coverage of the identified sample?** All studies were classified as ‘Not applicable’ because most (if not all) of them are based on secondary data and this information is usually presented by the responsible for the data collection process.

**Was the condition measured in a standard, reliable way for all participants?** All studies were classified as ‘Not applicable’ because most (if not all) of them are based on secondary data and it is uncommon for studies to discuss the validity of the data collection methods of the data sources.

**Was there appropriate statistical analysis?** All studies not reporting uncertainty measures were classified as “no” based on the criteria definition.

**Was the response rate adequate, and if not, was the low response rate managed appropriately?** All studies were classified as ‘Not applicable’ because most (if not all) of them are based on secondary data and it is uncommon for studies report the response rate of their data sources, especially when multiple data sources are utilized.

# Distribution of reviewed studies over time
